# Quercetin alleviates pulmonary angiogenesis in a rat model of hepatopulmonary syndrome

**DOI:** 10.1590/1414-431X20165326

**Published:** 2016-07-04

**Authors:** X. Li, Y. Chen, L. Wang, G. Shang, C. Zhang, Z. Zhao, H. Zhang, A. Liu

**Affiliations:** 1 Department of Physiology, Changzhi Medical College, Changzhi, China; 2 Department of Microbiology, Changzhi Medical College, Changzhi, China; 3 Functional Laboratory of Changzhi Medical College, Changzhi, China; 4 Liver Disease Institute of Changzhi Medical College, Changzhi, China; 5 Institute of Chinese Materia Medica, China Academy of Chinese Medical Sciences, Beijing, China

**Keywords:** Angiogenesis, Hepatopulmonary syndrome, HIF-1α, Quercetin

## Abstract

Quercetin shows protective effects against hepatopulmonary syndrome (HPS), as demonstrated in a rat model. However, whether these effects involve pulmonary vascular angiogenesis in HPS remains unclear. Therefore, this study aimed to assess the effect of quercetin on pulmonary vascular angiogenesis and explore the underlying mechanisms. Male Sprague-Dawley rats weighing 200-250 g underwent sham operation or common bile duct ligation (CBDL). Two weeks after surgery, HIF-1α and NFκB levels were assessed in rat lung tissue by immunohistochemistry and western blot. Then, CBDL and sham-operated rats were further divided into 2 subgroups each to receive intraperitoneal administration of quercetin (50 mg/kg daily) or 0.2% Tween for two weeks: Sham (Sham+Tween; n=8), CBDL (CBDL+Tween; n=8), Q (Sham+quercetin; n=8), and CBDL+Q (CBDL+quercetin; n=8). After treatment, lung tissue specimens were assessed for protein (immunohistochemistry and western blot) and/or gene expression (quantitative real-time PCR) levels of relevant disease markers, including *VEGFA*, VEGFR2, Akt/p-Akt, HIF-1α, vWf, and IκB/p-IκB. Finally, arterial blood was analyzed for alveolar arterial oxygen pressure gradient (AaPO_2_). Two weeks after CBDL, HIF-1α expression in the lung decreased, but was gradually restored at four weeks. Treatment with quercetin did not significantly alter HIF-1α levels, but did reduce AaPO_2_ as well as lung tissue NF-κB activity, *VEGFA* gene and protein levels, Akt activity, and angiogenesis. Although hypoxia is an important feature in HPS, our findings suggest that HIF-1α was not the main cause for the VEGFA increase. Interestingly, quercetin inhibited pulmonary vascular angiogenesis in rats with HPS, with involvement of Akt/NF-κB and VEGFA/VEGFR-2 pathways.

## Introduction

Hepatopulmonary syndrome (HPS) is a serious pulmonary microvascular complication that causes systemic hypoxemia in the setting of liver disease. It occurs in up to 32% of patients with cirrhosis and significantly increases mortality (1,2). Common bile duct ligation (CBDL) in rats is a classical model that mimics the pathological process of human HPS (3,4). Similar to human HPS, besides the abnormal pulmonary microvascular vasodilatation, rats with CBDL also show angiogenesis (5). Several lines of evidence suggest that angiogenesis is one of the critical events inducing hypoxemia in experimental HPS, and vascular endothelial growth factor A (VEGF-A) is involved in the process (5-8).

Hypoxemia is an important feature of HPS; meanwhile, hypoxia-inducible factor-1 (HIF-1)α is commonly induced in hypoxic conditions, and is regulated by endothelin, a key effector in HPS ([Bibr B09]9-11). We hypothesized that HIF-1α may play an important role in the pathogenesis of HPS as well. Therefore, we assessed HIF-1α levels in the CBDL model. VEGF-A, a pivotal regulator of angiogenesis, is secreted by many types of cells, including endothelial cells and macrophages, and is mainly regulated by HIF-1 under hypoxic conditions; therefore, HIF-1α regulatesthe VEGF signaling pathway (12-14). Previous studies have revealed that VEGF-A production by pulmonary intravascular macrophages and pulmonary vascular endothelium cells contributes to intrapulmonary angiogenesis and intrapulmonary shunts, which leads to abnormal gas exchange and induction of hypoxia in CBDL rats (5,7,8). These findings suggested that targeting the VEGF-A signaling pathway may constitute a promising anti-angiogenic therapy for the treatment of HPS. It is also known that PI3K/Akt signaling is a key pathway in HPS associated pulmonary angiogenesis (13), and it might regulate HIF-1α and NF-кB signaling (15).

Quercetin (3,5,7,3-4-pentahydroxy flavone), a plant flavonoid present in various foods, exerts protective effects in rats with HPS via different mechanisms including reduction of oxidative stress and modulation of NF-κB signaling pathways (16,17). Previous studies have highlighted the protective effect of quercetin in pulmonary complications of liver disease (16,17). Recently, quercetin has attracted increasing attention because of its pro-angiogenic or anti-angiogenic effect. Some studies reported the anti-angiogenic effects of quercetin on tumor, choroidal, and retinal angiogenesis *in vivo* and *in vitro* (18-21). In addition, quercetin activates an angiogenic pathway in colonic mucosal injury (22). However, it remains unknown whether quercetin protects against HPS by modulating pulmonary vascular angiogenesis. Therefore, we aimed in this study to assess the effects of quercetin on pulmonary vascular angiogenesis and explore the potential involvement of Akt/NF-κB and VEGFA/VEGFR-2 pathways, which play an important role in HPS pathogenesis.

## Material and Methods

### Animal protocol

The Institutional Animal Care and Use Committee of Changzhi Medical College approved all experimental protocols. Male Sprague-Dawley rats weighing 200-250 g were obtained from Beijing Vital River Laboratory Animal Technology Co. Ltd. (China) and provided with unrestricted chow and water. In the first experiment, 16 rats underwent sham operation or CBDL under ketamine anesthesia (100 mg/kg of body weight, intramuscular), as previously described (5-8). Two weeks after surgery, HIF-1α expression was assessed in rat lung tissue specimens by immunohistochemistry (IHC) and western blot. In another experiment, a total of 32 additional animals were divided into Sham (Sham+Tween; n=8), CBDL (CBDL+Tween; n=8), Q (Sham+quercetin; n=8), and CBDL+Q (CBDL+quercetin; n=8). Sham and Q groups underwent sham operation while the CBDL and CBDL+Q groups were submitted to CBDL. Two animals died in the CBDL group, leaving 6 animals that were assessed (Supplementary Figure S1). Two weeks after surgery, the Q and CBDL+Q groups were administered daily quercetin (50 mg/kg, dissolved in 0.2% Tween) intraperitoneally (16,17) for 2 weeks, while the Sham and CBDL control groups received equivalent volumes of 0.2% Tween for the same duration. Lung tissue specimens were harvested from each animal, rinsed in cold isotonic saline, and either fixed with 4% paraformaldehyde or immediately frozen in liquid nitrogen for further analysis.

### Arterial blood gas analysis

Four weeks after surgery, arterial blood was withdrawn from the femoral artery, and analyzed on an ABL 520 radiometer (Radiometer America, USA). The alveolar arterial oxygen pressure gradient (AaPO_2_) was calculated as 150 − (PaCO_2_/0.8) − PaO_2_, according to previous reports (5,7).

### Immunohistochemistry and immunofluorescence

Lung tissue sections (4-μm thick) were prepared from paraffin-embedded specimens, using conventional methods. Endogenous peroxidase was inactivated by treating slides with 3% H_2_O_2_ at room temperature for 15 min, followed by antigen retrieval. After blocking with goat serum (BSA) for 30 min, rabbit anti-HIF-1α (1:200, wl01607, Wanleibio, China) primary antibody was added for overnight incubation at 4°C. The slides were then incubated with a secondary antibody for 1 h at room temperature. Detection was carried with the DAB reagent, and slides were counterstained with hematoxylin, dehydrated, and mounted for subsequent microscopic examination. Alternatively, rabbit anti-vWF polyclonal primary antibody (1:300, ab6994, Abcam, USA) was used for immunofluorescence, while rabbit anti-NF-κB p65 polyclonal antibody (1/1000, ab16502, Abcam) and mouse monoclonal anti-VEGF antibody (VG-1) (1:100 ab1316, Abcam) were employed for double immunofluorescence labeling, followed by addition of corresponding fluorescent-labeled secondary antibodies, goat anti-Rabbit IgG H&L (FITC) (1:2000, ab6717, Abcam) or goat anti-mouse IgG H&L (Alexa Fluor^¯^ 647; 1:600, ab150115, Abcam) secondary antibodies. The slides were then incubated at room temperature for 1 h and mounted in the DAPI-containing mounting medium for microscopic examination. Microvascular density was assessed as previously described (23). Positive HIF-1α signals were quantitated with the Image-Pro Plus7.0 software (Media Cybernetics, USA).

### Quantitative real-time PCR

Total RNA from lung tissue specimens was extracted with TRIzol reagent, and reverse transcribed into cDNA using PrimeScript^¯^ RT Reagent Kit (TaKaRa, China). Real-time PCR was performed on a MyiQ2 two-color real-time PCR detection system (model IQ5; Santa Cruz Biotechnology Co., Ltd., China), according to the manufacturer's recommendations. The following primers for *VEGF-A* were used: sense 5′-CGTCTACCAGCGCAGCTATTG-3′; anti-sense, 5′-CACACAGGACGGCTTGAAGAT-3′. Data were normalized to 18S rRNA gene expression levels (sense, 5′-CGGCTACCACATCCAAGGAA-3′; anti-sense, 5′-GCTGGAATTACCGCGGCT-3′). Relative gene expression levels were calculated by the 2^−ΔΔ^ ct method.

### Western blot analysis

Lung tissue homogenates were lysed to extract total protein, quantified by the bicinchoninic acid assay (BCA) method. Fifty micrograms of total protein per sample were separated by SDS-PAGE and electro-transferred onto a nitrocellulose membrane. After blocking, primary antibodies raised against VEGFA (1:800, WL00009b, Wanleibio), VEGFR2 (1:300, sc-504; Santa Cruz Biotechnology Co., Ltd.), p-VEGFR2 (1:200 sc-16629-R Santa Cruz), Akt (1:500, 4691s; Santa Cruz), P-Akt (Ser473, 1:500, 4060 s; Cell Signaling Technology, USA), HIF-1α (1:800, 20960-1-AP; ProteinTech Group), β-actin (1:5000, BM0005; Boster, China), p-IκBα (1:1000; 2859, Cell Signaling Technology), and IκBα (1:1000, 9242; Cell Signaling Technology) were added, respectively, for overnight incubation at 4°C. After washing, the corresponding horseradish peroxidase-labeled secondary antibodies were added for 1.5 h at room temperature. Enhanced chemiluminescent detection was performed, followed by exposure to X-ray film and development. β-actin was used as an internal reference. Protein bands were quantitated with the Image-Pro Plus 7.0 software.

### Statistical analysis

The SPSS (IBM, USA) 19.0 software was applied for all statistical analyses. Data were assessed by one-way analysis of variance, with *post hoc* Tukey's test. Data are reported as means±SD. P<0.05 was considered to be statistically significant.

## Results

### Pathological features of human HPS in CBDL rats

**Figure 1:**
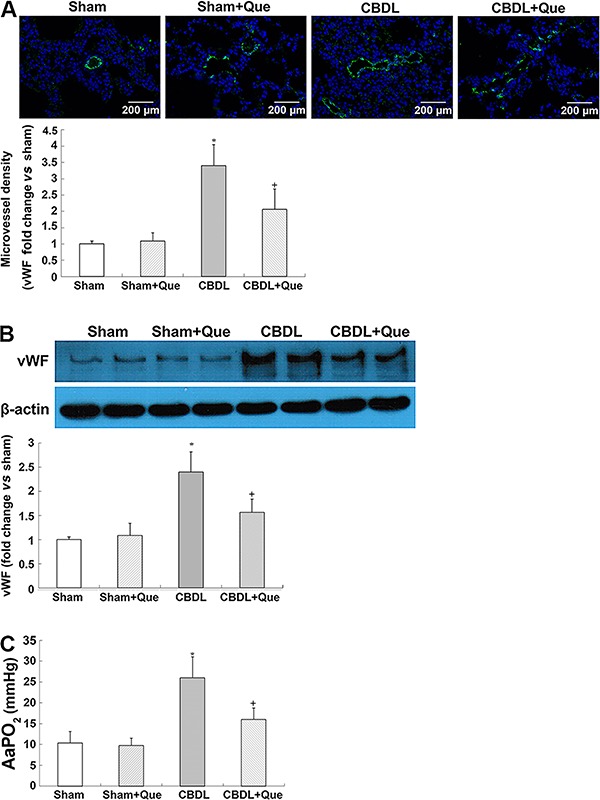
Effects of quercetin on pulmonary von Willebrand Factor (vWF) immunostaining levels, alveolar-arterial oxygen gradients, and portal hypertension and hepatic fibrosis in common bile duct ligation (CBDL) rats, sham-operated rats (Sham), Sham+quercetin (Que) rats, and CBDL+quercetin (CBDL+Que) rats. *A*, immunostaining of vWF (green) with DAPI nuclear stain (blue) and lung microvessel density. (*B*), pulmonary expression of vWF protein levels and lung microvessel density. *C*, summary of alveolar arterial oxygen pressure gradient (AaPO_2_) in all groups. Sham, n=8; CBDL, n=6; Que, n=8; CBDL+Que, n=8. Data are reported as mean ±SD. *P<0.05 compared with sham rats. ^+^P<0.05 compared with CBDL rats (ANOVA).

The key characteristics of human HPS, including increased lung microvessel density and AaPO_2_, were observed in CBDL rats. Interestingly, quercetin treatment markedly attenuated vWF-positive pulmonary microvascular signals and decreased vWf protein levels, indicating an amelioration of pulmonary angiogenesis. Meanwhile, alleviation of hypoxemia in HPS rats after quercetin treatment was observed, as manifested by decreased AaPO_2_ (Figure 1A, B, and C).

### Expression of VEGF-A, VEGFR-2, and phospho-VEGFR-2 in the pulmonary tissue of CBDL rats

**Figure 2:**
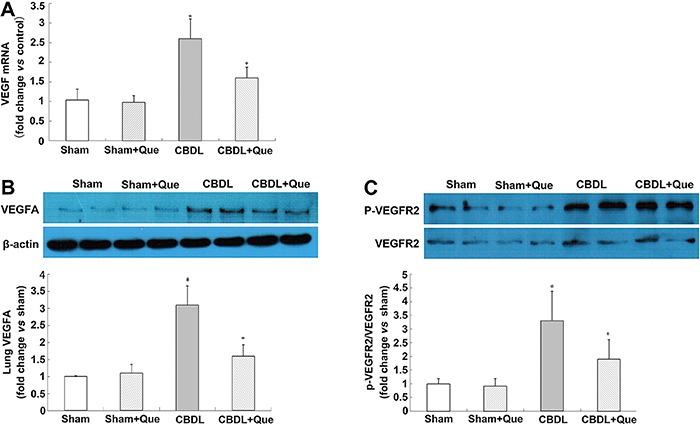
Effect of quercetin on mRNA and protein expression of lung VEGF-A, VEGFR2 and P-VEGFR2 in all rats (*A*), lung *VEGF-A* mRNA levels representative immunoblots and graphical summaries of lung VEGF-A protein levels (*B*), representative immunoblots and graphical summaries of lung VEGFR2 and P-VEGFR2 (*C*). Sham: sham-operated, n=8; CBDL: common bile duct ligation, n=6; Que: Sham+quercetin, n=8; CBDL+Que, n=8. Data are reported as mean ±SD. *P<0.05 compared with sham rats. ^+^P<0.05 compared with CBDL rats (ANOVA).

To explore the mechanisms of quercetin in pulmonary angiogenesis, mRNA and protein levels of VEGF-A were assessed, and found to be significantly decreased after quercetin treatment. The protein levels of VEGFR-2 and p-VEGFR-2 were also assessed. Although no significant alteration was found in VEGFR-2, p-VEGFR-2 was significantly decreased after quercetin treatment (Figure 2A, B, and C).

### Expression of Akt and p-Akt in the pulmonary tissue of CBDL rats

**Figure 3:**
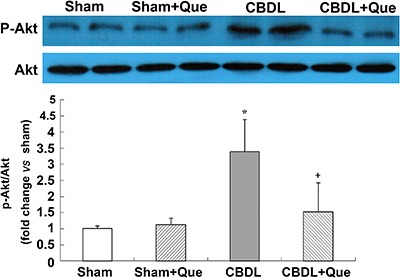
Effects of quercetin on lung Akt activation after common bile duct ligation (CBDL). Representative immunoblots and graphical summaries of lung p-Akt/Akt. Sham: sham-operated, n=8; CBDL: common bile duct ligation, n=6; Que: Sham+quercetin, n=8; CBDL+Que, n=8. Data are reported as mean ±SD. *P<0.05 compared with sham rats. ^+^P<0.05 compared with CBDL rats (ANOVA).

The PI3K/Akt pathway mediates VEGF-induced pulmonary angiogenesis. To evaluate the activation of pulmonary PI3K/Akt signaling in CBDL rats, protein levels of p-Akt (the active form of Akt) were measured by western blotting. Interestingly, the p-Akt protein levels that were significantly increased after CBDL, were substantially reduced by treatment with quercetin (Figure 3).

### Expression of HIF-1α in the pulmonary tissue of CBDL and sham-operated rats

**Figure 4:**
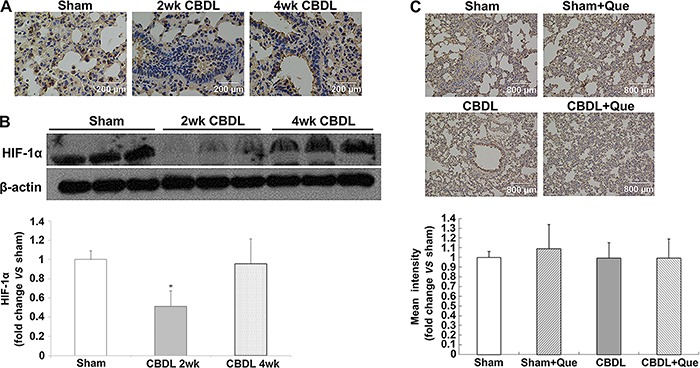
Pulmonary HIF-1α expression and localization after common bile duct ligation (CBDL). *A*, Representative images of lung immunohistochemistry for HIF-1α 2 and 4 weeks after CBDL (original magnification, 40×). *B*, Representative immunoblots and graphical summaries of lung HIF-1α 2 and 4 weeks after CBDL. *C*, Immunohistochemical staining to assess the effects of quercetin on pulmonary HIF-1α expression and localization (original magnification, 10×) and its graphical representation. For the 2-weeks time: Sham (sham-operated), n=8; CBDL, n=8. For the 4-weeks time: Sham, n=8; CBDL, n=6; Que (sham+quercetin), n=8; CBDL+Que, n=8. Data are reported as the mean±SD. *P<0.05 compared with sham rats (ANOVA).

HIF-1α is the major regulator of VEGF at the transcriptional level. To assess whether HIF-1α is also involved in pulmonary angiogenesis in rats with HPS, localization and expression of HIF-1α were evaluated by IHC and western blotting at 2 and 4 weeks after surgery. As shown in Figure 4, pulmonary HIF-1α levels were markedly decreased two weeks after CBDL and restored at 4 weeks. Of note, quercetin did not reduce HIF-1α accumulation in sham and HPS rats (Figure 4A, B, and C).

**Figure 5 f05:**
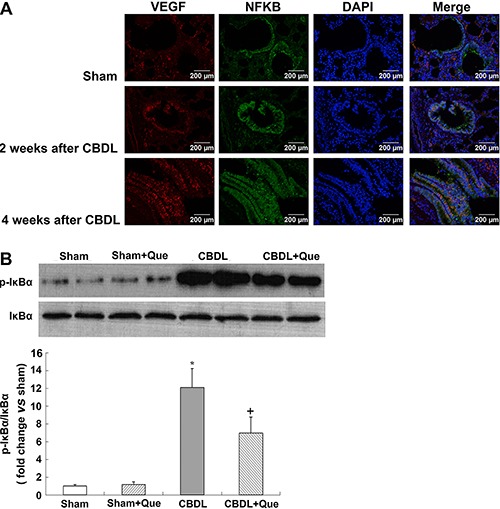
Immunofluorescence localization of NF-κB p-65 and VEGF-A after common bile duct ligation (CBDL). Effects of quercetin (Que) on the level of lung IκBα phosphorylation after CBDL. *A*, Representative double-labeling images of p-65 and VEGF-A (original magnification, 40×). *B*, Representative immunoblots and graphical summaries of lung p-IκBα/IκBα. Sham (sham-operated), n=8; CBDL, n=6; Que (sham+quercetin), n=8; CBDL+Que, n=8. Data are reported as the mean±SD. *P<0.05 compared with sham rats. ^+^P<0.05 compared with CBDL rats (ANOVA).

### NF-κB activation in the pulmonary tissue of CBDL and sham-operated rats

In addition to HIF-1, the expression of VEGF is also stimulated by NF-κB (24). Here, we evaluated the co-localization and expression of NF-κB and VEGF-A by double immunofluorescence labeling, and evaluated NF-κB activation in the lung tissue by western blotting. In the normal lung, minimal NF-κB and VEGF-A staining was observed. Four weeks after CBDL, NF-κB staining was markedly increased in rats, accompanied by increased VEGF-A signals. Interestingly, quercetin treatment significantly reduced NF-κB and VEGF-A signals. Western blotting showed that phosphorylation of NF-κB inhibitory protein IκBα was significantly increased in untreated CBDL rats, indicating increased NF-κB activity; quercetin treatment significantly lowered the levels of pulmonary p-IκBα.

## Discussion

This study assessed whether the protective effects of quercetin against HPS involved pulmonary vascular angiogenesis. Interestingly, we demonstrated that quercetin inhibited pulmonary vascular angiogenesis in rats with HPS, via the Akt/NF-κB and VEGF-A/VEGFR-2 pathways.

Pulmonary microvascular angiogenesis has been linked to the development of HPS as an important contributor to hypoxemia (5,7). Although liver transplantation is currently deemed to be a successful therapy for HPS, some liver recipients show no improvement in symptoms such as hypoxia and impaired gas exchange capacity (24-26). This could be attributed to the pulmonary microvascular angiogenesis in patients with HPS (25,26). Therefore, it is important to detect and control pulmonary angiogenesis in patients with HPS. In this study, we used CBDL rats to simulate human HPS, specifically focusing on the effects of quercetin on pulmonary microvascular angiogenesis. vWf, a specific marker for endothelial cells, was quantitated by IHC staining or western blot analysis to assess vascularization in the pulmonary tissue. The decreased vWF+ vessels and vWf protein levels suggest that quercetin treatment attenuates pulmonary angiogenesis in CBDL rats. Inhibiting angiogenesis this way can markedly improve hypoxemia (5,7,8,13). In agreement with a previous report (17), a significant improvement in hypoxemia was also observed in quercetin-treated CBDL rats.

Cumulative evidence supports the importance of VEGF-A signaling, including downstream Akt (protein kinase B) in CBDL rats with regard to pulmonary angiogenesis (5,7,13). Binding of VEGF to its receptor VEGFR-2 contributes to homo-dimerization and auto-phosphorylation of VEGFR-2, subsequently activating PI3K/Akt signaling. (27-29). Activated Akt is essential for the proliferation, survival, and migration of endothelial cells and for capillary tube formation (12). In this study, we found that quercetin administration not only downregulated the expression of pulmonary VEGF-A but also significantly decreased p-VEGFR-2 and p-Akt protein levels in the CBDL rats. Multiple reports (30-32) demonstrated that hypoxia or hypoxemia cause VEGFR-2 phosphorylation, and subsequently, neovascularization. Taken together, our findings indicate that quercetin exerts its anti-angiogenic effect through inhibition of the VEGF/VEGFR-2/Akt pathway in rats with HPS.

Interestingly, PI3K/Akt signaling can also regulate the expression of VEGF-A by regulating HIF-1α (33,34). HIF-1α is an active subunit of HIF-1. Under hypoxic conditions, the HIF-1α protein stabilizes and translocates from the cytoplasm into the nucleus, where along with HIF-1β, it forms transcriptionally active HIF-1 heterodimers, thereby activating the transcription of VEGF, which is then expressed and secreted (35). Several studies have shown that the PI3K/Akt pathway can promote the stabilization and nuclear accumulation of HIF-1α (36,37). We can deduce that the overexpression of phospho-Akt increases the level of HIF-1α in rats, subsequently increasing VEGF expression. However, in our study, we observed decreased expression and nuclear accumulation of HIF-1α in the pulmonary tissue of rats with HPS after 2 weeks of CBDL. After 4 weeks of CBDL, HIF-1α levels in the CBDL rats were restored to levels similar to those in normal controls, and the difference was not statistically significant. IHC analysis of the lung tissue showed that the expression and nuclear accumulation of HIF-1α did not differ significantly between untreated CBDL rats and quercetin-treated CBDL rats. Taken together, these results suggest that hypoxia was not the main reason for the increased VEGF and angiogenesis in CBDL rats, and that after quercetin treatment, reduced pulmonary angiogenesis was not via the HIF-1α/VEGF pathway. To our knowledge, HIF-1α was firstly detected here in the context of HPS. Interestingly, recent reports suggested that bilirubin inhibits HIF-1α activation, while CBDL causes increased bilirubin levels (38,39); this might, at least in part, explain our findings of reduced HIF-1α levels in CBDL animals.

In addition to HIF-1, NF-κB has also been reported to upregulate VEGF expression and promote angiogenesis (40,41), and PI3K/Akt pathway has also been implicated in the regulation of NF-κB (42). Previous studies have shown that Akt-dependent phosphorylation of IκB protein leads to degradation of IκB and dissociation from NF-κB, thereby resulting in NF-κB activation and migration to the nucleus. In the nucleus, active NF-κB binds to the specific promoter of *VEGF* gene, and therefore initiates VEGF transcription (43,44) increases VEGF-A expression, and promotes vascular angiogenesis. In the present study, the protein levels of p-Akt and p-IκB simultaneously increased after CBDL, along with elevated VEGF-A expression. IHC analysis of the lung tissue showed that the expression and distribution of VEGF-A is correlated with that of NF-κB. In view of the role of the PI3K/Akt/NF-κB pathway in VEGF production, our results suggest that NF-κB activation increased angiogenesis as a result of elevated VEGF-A levels in CBDL rats. Previous studies have revealed that quercetin can inhibit NF-κB activation in rats with HPS (17). Here, we observed that quercetin treatment resulted in decreased Akt activation in CBDL rats and simultaneously reduced p-IκB level, which indicated a lower activity of NF-κB in lung tissues. Taken together, our results indicated that quercetin can decrease VEGF-A expression in the lung tissues of rats with HPS, possibly via the PI3K/Akt/NF-κB pathway, and can reduce pulmonary vascular angiogenesis.

In this study, we did not evaluate the effects of quercetin on the liver. Several studies have reported the effects of quercetin on the liver in this model. In addition, we believe that the correlation of severity between the HPS and liver disease is not reliable (45). In addition to this model, neither HPS nor pulmonary vascular angiogenesis was observed in various other models with severe liver cirrhosis (5). Furthermore, quercetin treatment could not reopen the common bile duct of CBDL rats. This suggests that quercetin not only acts on the liver but also on lung tissues to reduce pulmonary microvascular angiogenesis.

In conclusion, this study demonstrated the anti-angiogenic effects of quercetin on pulmonary tissue in CBDL rats. The mechanism underlying these effects in HPS might be partly occurring via Akt/NF-κB pathway inactivation, but not HIF-1α, and via attenuation of the VEGF-A/VEGFR-2 related angiogenesis pathway.

## Supplementary material

Click here to view [http://bjournal.com.br/supplementary_material/5326.pdf].
